# Strengthening respectful communication with patients and colleagues in neonatal units — developing and evaluating a communication and emotional competence training for nurse managers in Kenya

**DOI:** 10.12688/wellcomeopenres.18006.2

**Published:** 2025-04-25

**Authors:** Peris Musitia, Mwanamvua Boga, Dorothy Oluoch, Ane Haaland, Jacinta Nzinga, Mike English, Sassy Molyneux

**Affiliations:** 1 The Aga Khan University Hospital Nairobi, Nairobi, Nairobi County, Kenya; 2 Health Service Unit, KEMRI Wellcome Trust Research Programme, Nairobi, Kenya; 3 Institute of Health and Society, Faculty of Medicine, University of Oslo, Oslo, Norway; 4 Liverpool School of Tropical Medicine, Liverpool, England, UK; 5 Nuffield Department of Medicine, Oxford Centre for Global Health Research, Oxford, Oxford, UK

**Keywords:** Emotional competence, nurse well-being, management, reflective practice, communication, relationships, quality of care

## Abstract

**Introduction:**

Effective communication is essential to delivering compassionate, high-quality nursing care. The intensive, stressful and technical environment of a new-born unit (NBU) in a low-resource setting presents communication-related challenges for nurses, with negative implications for nurse well-being, team relationships and patient care. We adapted a pre-existing communication and emotional competence course with NBU nurse managers working in Kenya, explored its’ value to participants and developed a theory of change to evaluate its’ potential impact.

**Methods:**

18 neonatal nurse managers from 14 county referral hospitals helped adapt and participated in a nine-month participatory training process. Training involved guided ‘on the job’ self-observation and reflection to build self-awareness, and two face-to-face skills-building workshops. The course and potential for future scale up was assessed using written responses from participant nurses (baseline questionnaires, reflective assignments, pre and post workshop questionnaires), workshop observation notes, two group discussions and nine individual in-depth interviews.

**Results:**

Participants were extremely positive about the course, with many emphasizing its direct relevance and applicability to their daily work. Increased self-awareness and ability to recognize their own, colleagues’ and patients’ emotional triggers, together with new knowledge and practical skills, reportedly inspired nurses to change; in turn influencing their ability to provide respectful care, improving their confidence and relationships and giving them a stronger sense of professional identity.

**Conclusion:**

Providing respectful care is a major challenge in low-resource, high-pressure clinical settings but there are few strategies to address this problem. The participatory training process examined addresses this challenge and has potential for positive impacts for families, individual workers and teams, including worker well-being. We present an initial theory of change to support future evaluations aimed at exploring if and how positive gains can be sustained and spread within the wider system.

## Introduction

Effective communication is essential to the delivery of compassionate, high quality nursing care. Emotional competence - the ability to perceive, understand and manage emotions and to understand and relate effectively to others - is central to effective communication ^1, 2^. The intensive, stressful and technical environment of a new-born unit in a low resource setting can be emotionally fraught for parents and staff, presenting particular communication challenges for nurses ^3^.

Effective communication between nurses and patients is characterized as open, two-way communication in which patients and their family members learn about their illness and are encouraged to express their anxieties and feelings ^4^. Multiple studies have linked effective communication to better patient outcomes, such as improved patient satisfaction, understanding, and adherence to therapy ^5, 6^. An important influence on communication between nurses and patients and family members is the nurses’ work environment, including their relationships with colleagues and supervisors, workloads and access to resources ^7, 8^. Effective communication among nurses has been shown to create safer work environments, to build mutual respect, trust and understanding within teams, and to support decision making ^9^.

Many public sector facilities in sub-Saharan Africa face significant resource constraints, with important implications for nurses’ emotional well-being and communication. High workloads, low staffing levels, resource shortages, and environmental inadequacies (crowding, lack of privacy) contribute to the high levels of stress and ‘burnout’ ^10^. Stress and burnout negatively affect relationships with colleagues, patients and family members, which undermines motivation, team work, and ultimately quality of care ^11^. A recent review of communication focused on sub-Saharan Africa most often noted positive practice in HIV, intensive and palliative care settings, and in operative/post-operative care ^12^. However the authors noted strong power asymmetries between patients and nurses across all geographical and clinical settings, contributing to a lack of information sharing and open discussion, and even – in some antenatal care settings – leading to nurses abusing patients or denying them needed care ^12^. 

In new-born units (NBUs) specifically, there are particular challenges for communication ^3, 7^. The admission of a baby into a neonatal unit can lead to severe maternal distress, with some mothers describing the experience as devastating, traumatic and life-altering ^7, 13^. Mothers are often exhausted and anxious about their babies’ health and well-being, concerned by the machinery and equipment they see around them and can be unwell and in pain themselves ^7^. In these intensive, stressful environments, nurses’ communication is expected to go beyond informing parents of the neonate’s clinical condition. They are also often expected to reassure and empathise with parents, educate and guide them on the care of their babies, support mothers in bonding with their babies, and encourage parents to participate in decision-making ^14, 15^. However, in a cross-sectional observational study in Kenya this was rarely observed to happen in practice ^3^. Also in Kenya, Brown noted in her ethnographic work that mothers were often given nurses’ care-giving tasks, but without the informational and emotional support they needed ^16^. In South Africa, such challenges contributed to mothers being anxious and unwilling to ask questions, leading to nurses feeling that parents did not want to be engaged which acted as a barrier in mothers’ participation in their baby’s care ^7^.

The importance of communication skills and emotional competence training is increasingly recognised for health professionals working in sub-Saharan Africa, but few studies have documented the implementation and impact of training ^10^. A systematic review of communication skills training for health professionals suggests that most programs last 4½ hours to 2 days, and include a combination of lectures, videos and dramatizations ^17^. The review concludes that training in communication skills can improve health professionals’ performance and their self-efficacy (their belief in their ability to perform a task successfully), and that programmes are most successful where space for experiential learning is incorporated. Even short courses on communication skills and emotional competence have been reported to increase job satisfaction and reduce work stress ^18– 20^, with recommendations for training including: ensuring that knowledge and skills taught are contextualized to participants’ working realities; promotion of affective changes that motivate and provoke the desire to use the new knowledge and skills; and implementing courses over time (rather than as once off short interventions) ^21^.

The work presented here has its origin in the Intelligent Communication, Awareness and Action, Reflection, Emotions model (the iCARE-Haaland model) developed and implemented by Ane Haaland in collaboration with physicians and nurses in nine countries over almost two decades, including Kenya. The original model was built to address TB and HIV. In Kenya, AH worked with Mwanamvua Boga (MB) to broaden the content to cover other diseases, using the same reflective and experiential learning methods. The model, its' development and all associated tools are available from The Global Health Network website. The aim of the iCARE-Haaland Model is building health professionals’ capacity to build safe and trusting relationships, provide patient-centred care, communicate and relate well with colleagues, build emotional competence and take better care of their own health and wellbeing. The course includes a special emphasis on using reflective practice to support self-awareness and build personal and professional relationships using participatory principles ^22, 23^.

In this paper we describe how we adapted this pre-existing communication and emotional competence course for new born nurse managers working in public hospitals in Kenya. We examine the course value to participants and its’ potential for impact (findings). In the discussion we draw on our findings and the literature to propose a theory of change regarding the potential of this intervention to impact positively on individuals and broader systems.

## Methods

We conducted a process evaluation of the participatory course drawing primarily on qualitative methods ^24^. Our overall aim was to describe the rollout of the adapted intervention and to explore the course value to participants and its’ potential for positive impact.

### Study setting and research team

The work presented in this paper was conducted within the clinical information network (CIN) ^25^. CIN is a collaboration between county hospitals (equivalent large Kenyan district general hospitals), researchers, the Ministry of Health and pediatric professional groups. It was established to improve data on the quality and outcomes of inpatient pediatric and newborn care and use these data to learn how to improve practice. The research team for this paper include the lead course trainer (MB), and health systems/clinical researchers who are either from Kenya or who had lived and worked in Kenya for several decades at the time of the research. Two of the research team members (JN and ME) were centrally involved in establishing and evaluating the CIN, while three were more independent (PM, DO and SM). AH designed the original iCARE-Model drawn on so centrally in this training. The work presented in this paper is the first step in a planned cascading of the training through a Trainer of Trainers process.

### Course participants

Purposive sampling was used to select nurses for course participation. The nurse participants were senior nurses in leadership positions, selected given their important and complex roles as hybrid managers with both managerial and clinical care roles ^26^. All NBU nurse managers working in the 14 CIN hospitals located across Nairobi, Central, Eastern and Western regions of Kenya were invited to participate. Thus, the nurses included in this course already had regular interactions with the CIN team. The communication training was designed to complement existing efforts to improve care within the selected hospitals. A total of 18 managers participated in the course, but two moved out of the neonatal units during the course, and were replaced by two new participants. In this paper we only draw on data from the 16 managers who completed the entire course process. 

### Adapting and co-creating the course

Adaptation was built into the original iCARE-Haaland model, and much of training process is intended to be ‘on the job’, with two workshops to support face to face learning. MB (joint first author) adapted the course with the nurse manager participants and was the lead trainer in implementation. She is a senior Kenyan NBU nurse manager herself, contributed to the expansion of the iCARE-Haaland Model after its’ original design by AH, and has led many nurse training projects in other Kenyan settings and in the Gambia.

**Figure 1.**
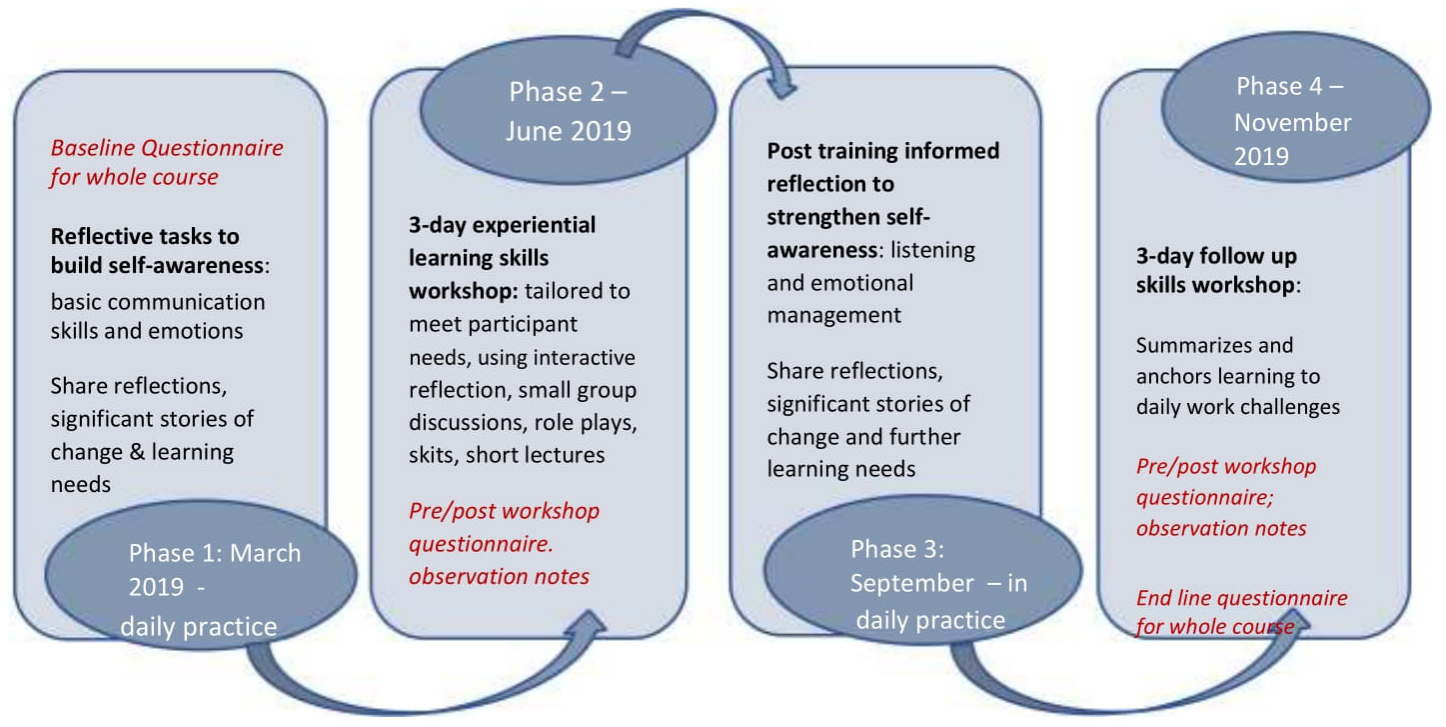
Describes the course process, course organization and course activities.

To adapt/tailor the course to the context, participant needs and ideas were explored through a one-day face-to-face introduction and planning session. The planning session aimed at understanding the challenges faced by nurses in communication with their colleagues and with patients. This planning session happened at a central place in Nairobi (capital city of Kenya) in 2019 and included 18 NBU nurse managers from CIN, mostly female (n=17) aged 30 to 55 years. All had at least six years nursing experience; 14 had more than 10 years’ nursing experience. The course used the adult learning approaches outlined in the iCARE Haaland model including role plays, demonstrations and group discussions to stimulate participants participation in the training. The phases and lengths of the adapted course are summarised in Figure 1, with more information on content provided in Table 1.

**Table 1.** Adapted Course process and course content.

Phase	Activities/content covered	Data incorporated into course
Phase 1: March -June 2019 **Discovery** **phase**	a) One-day planning meeting to understand challenges and needs b) Self-observation and reflection ‘on the job’ to discover own communication behaviours and effects on others (guided weekly tasks) to strengthen self- awareness and initiate a change process. **Reflective assignments pack 1 (3 weeks) : Basic communication** **skills** • **Week 1: Listening:** How well do you listen to your patients, colleagues, others and effects? • **Week 2: Asking questions:** How do you discuss, and ask questions when dealing with patients, colleagues and others. • **Week 3: Discussion habits:** When discussing with patients and colleagues, do you hinder or inspire good communication **Reflective assignments pack 2: Handling emotions over one** **month** observing own emotions, and those of patients and colleagues, and their effects; and reflecting on strategies they use to manage emotion	Filled post workshop open ended self-administered questionnaires in hand written notes Workshop observation notes
Phase 2 – June 2019 **Basic workshop** 3-day participatory skills workshop	A skills workshop tailored to participant needs and linking their self- observation and reflection to theory. The course adapted adult learning approaches by using role plays, demonstrations, group discussion and videos **Modules covered:** a) Adult learning theory, Basic communication skills b) Managing emotions with awareness c) Managing conflict to maintain dignity and respect for patients and colleagues. d) Principles of supportive supervision e) Dealing with death and dying	Submitted feed from the reflective assignment tasks in hand-written notes in their books and journal
Phase 3 -June -Oct 2019 **Skills into** **action**	Participants continued with another two sets of guided self-observation and reflective assignments to deepen and confirm learning. **Reflective assignments pack 3:** strengthening communication with colleagues **Reflective assignment pack 4:** communication with supervisors; taking care of safety, and of emotions.	Submitted feed from the reflective assignment tasks in hand-written notes in their books and journal
Phase 4 – November 2019 **Follow-up** **workshop** 3-day participatory skills workshop	A three day follow up skills building workshop to share learning and success stories in using the new skills, strengthening confidence and empowerment, and building new skills: **Modules covered** a) Review of communication strategies with colleagues b) What makes people change attitudes and behaviour? Why don’t patients change behaviours’? c) Why and how to recognize, manage and prevent stress and burnout d) The many phases of anger: Recognize, acknowledge and handle with respect. e) Dealing constructively with conflict: From confronting – to stepping back, and dialogue.	Filled pre- and post-course evaluation open ended self- administered questionnaire Workshop observation notes

Agreed amendments from the original course included reducing the main skills-building workshop from five to three days to better suit manager availability, and slightly shorter periods of focused self-observation in nurse managers’ normal workplaces in the months before and after the main workshop. Content of the course was adapted to include key concerns and issues for nurse managers, including dealing with difficult colleagues, managing staff conflicts linked to duty rotas, and handling patient deaths. Throughout the course, nurse managers’ own needs and examples, anonymised, fed into the materials of the following phases.

### Course process

Much of the learning was intended to be ‘on the job’, with only six days in total being face-to-face learning (two workshops, each of three days in June and November 2019) run by MB. Course participants were introduced to an initial set of reflective tasks, to support focused periods of self-observation in their normal workplaces over the three months before the first workshop as shown in Figure 1. The reflective assignments were drawn upon by MB to develop materials for the three-day initial training workshop and three-day skills building workshop which drew on experience based learning. The workshops were held in the capital city, Nairobi, facilitating relatively easy travel for all participants. The iCARE-Haaland model pre- and post-activity evaluations were built into course, including: a self-administered baseline questionnaire; a workshop evaluation drawing from anonymized diaries/transcriptions; and requests for participants to share ‘most significant change stories’ from reflective assignments. Tools are available on the iCARE-Haaland model website. The lead trainer (MB) received self-reflections by WhatsApp/ email during phases 1 and 3, and was available to offer input and support by phone as needed. Across all participants, this generally demanded one-two hours per week.

### Data collection

To explore the course value to participants and potential for positive impact and scale up, we drew on data collected as part of the course process, supplemented by specifically organised semi-structured individual and group interviews held in English and Kiswahili as preferred by the participants. Data collected as part of the course process included:

- An overall baseline and post intervention open ended questionnaire filled by all course participants (n=18 and 16 respectively), documenting their views on their own strengths and challenges in communicating with colleagues, supervisors and patients in neonatal care wards.
- Pre and post workshop open ended training questionnaires tracking participants’ views on the most valuable learning and relevance of the modules to their work.
- Written reflections submitted by participants (both hand written and typed reflections). This was written down in their daily journals, note books or diary (as part of Phase 1 and Phase 3).

To supplement the above data, PM (joint first author) observed the initial planning workshop and both skills-training workshops (Phases 2 and 4). These observations were invaluable given the very rich small group and plenary discussions organised throughout the workshops, and the opportunity to observe participant interest, enthusiasm and interactions. We also held individual and group interviews with course participants after the workshop. Two group discussions were organised with five participants before they left Nairobi, and an additional nine individual in-depth interviews were conducted with nurses once they had returned to their respective hospitals (November and December 2019). In-depth interviews were conducted until the researchers found that no new themes were being introduced. Interviewees were purposively selected to maximise diversity in potential perceptions of the course, based on observed level of engagement throughout the process, including whether or not they had completed their reflective assignments.

Data from these qualitative methods (reflective assignments, pre and post workshop open ended questionnaires and individual and group interviews) were triangulated to strengthen trustworthiness of the data and their interpretation. We continued to organise interviews until no new themes of relevance were emerging.

### Ethics approval and consent to participate

Ethics approval was obtained from the Kenya Medical Research Institute Scientific Ethics Review Committee (SERU) under number 3852. Permission to undertake the study was granted by the County department of health and the medical superintendents of each hospital. All participating nurses provided written informed consent for participation at the outset of the study and verbal consent to continue through all stages through to publication.

### Data management and analysis

Data were analysed using NVIVO 12 qualitative research data analysis software using a framework analysis approach which followed five steps of analysis: transcription, familiarization, coding creating and applying an analytical framework and charting ^27, 28^. All the digital recordings were transcribed verbatim by a team of external professional transcribers. PM read through the transcripts to gain understanding of the data before generating a coding framework. The initial framework, based both on the intended learning from the course and inductively from reviewing the transcriptions, was shared with the research team for input and agreement. The individual and group interview transcripts were first coded using this framework and later the data were condensed into matrix tables organised by themes agreed across the team to support data visualization, mapping and interpretation. Additional data from self-observation reflective notes and the observations were then coded and added into the matrices. These data also provided rich illustrations and examples, and some context to the coded data.

## Results

Following a brief summary of the overall perceived value of the course to the nurse participants, we outline the ways in which the course process fed into participants’ reported changes in their self-awareness and behaviour, including in relation to interactions with colleagues and parents of babies. 

### Overall value of the course

There was an overwhelmingly positive response to the course by participants, with many emphasising its direct relevance and applicability to their daily work in new born units, and the need for the course to be spread to others. Importantly, participants emphasised that the course was different to others they had participated in; that it was more powerful and inspiring of personal change. They talked in particular about the course having improved their self-awareness and ability to step back from automatic reactions, and about having changed their behaviour as a result.

*“[I realised that] when I shouted at the parent because of not following instructions, the parent feared me so much that she could not share with me anything, not even something to do with the patient and hence I could not give quality of care because I did not know more about the patient”. Nurse A, Reflection*
*“[Now] I am not just focusing on the sickness... [I] am looking at that mother wholesomely, because rather than the sickness that the mother is hav-ng, she is a complete mother. She is just like me, she has everything, so now am able to analyze other faculties her social status, mental status, yes.”* Nurse B, Reflection
*“Me, my most jaw breaker [learning] or what has helped me most, it is that I am now very patient. I used to be a really impatient person... I would want to do my tasks very fast, I do my perfection, I finish... [Now] I have learnt, I have all the patience especially to listen to the mothers. I'd say it has improved my relationship with my colleagues because of specifically the stepping back and awareness”.* Nurse C, Reflection

More generally participants described the course as assisting them with more positive interactions and relationships with peers, supervisors, and with patients’ family members, and that this in turn helped them manage other work stresses and challenges and overall feel more motivated.

*“I would say the course is very essential for nurses who are practicing the new born unit and interacting daily with the mothers of the new born babies, it simplifies the hurdles that are experienced in the new born unit daily, when you are interacting with the mothers or their relatives. It has made me now love my work more, to have no fear and be free even to ask for help from even seniors, from my supervisors; we are working without any fear.”* Individual Interview, Nurse, Facility 2
*“The moment you have undergone this training now you realize even your working space really changes, you have less conflicts, you have better satisfaction, you have less burn outs, you know actually when you go you can actually feel your outputs at your place of work, just because of that communication.*” Nurse Participant, Small group Discussion

Beyond the specific elements of the course feeding into change as outlined next, nurses also appeared to enjoy the face-to-face workshops where nurses with similar roles could network and discuss their shared experiences, concerns and managerial strategies in a safe space, and to build friendships.

### Self-observation and reflection strengthened self-awareness and motivation to change

An important element contributing to the perceived positive impact of the course was an increased awareness of one’s own communication style and limitations, initially developed through the reflection exercises. In these exercises, nurses kept a daily hand-written or typed up journal of their observations and reflections, and later shared their notes — if they were comfortable to do so — with the course facilitators. As two participants shared:

*“These few weeks of self-observation in my communication both to patients, relatives and my colleagues felt like I have a truth meter in myself. I have never been so conscious of what I spoke, how I respond and how my listening was affecting other people. And it felt like I am trying to balance myself on a weighing scale. But believe me or not it was all worthwhile. Every day I reflected on the day’s happenings, this made me understand that communication can change and affect the people we interact with.”* Participant Nurse, Reflection Tasks
*“They [self-observation and reflection tasks] are good, because they give me, as a learner, I get to understand myself better. I get to understand what I do wrong, what I do right, what I need to improve. So it’s important. It really boosts on the self-awareness.”* Individual Interview, Nurse, Facility 2

The first quote above in particular suggests that the self-awareness included a recognition at least among some participants that even in difficult environments, individuals themselves do have some power to make a difference, which may have motivated them to change their communication styles. Another participant highlighted how important self-reflection is over being told about ones’ limitations and faults by somebody else to motivate change:

*“The greatest difference I noted [from other courses], and I think it is a plus to the training is the reflection. The reflection, if you are sincere, it will gauge you. And you know if someone tells you are rude, you will be like ‘you are condemning me’ or it is like ‘you don’t know me’. But when you sit with yourself and see it, then you will pick it up because it is the truth. When I was given that opportunity to look at myself and the way I talk, the way I communicate, the way I lead, the way I behave, not only here, even at home [laughs] even in church, I was like okay, I need this course.”* Individual Interview, Nurse, Facility 9

### Training content reflected course participants’ shared experiences and needs

The observation and reflection tasks were designed to create awareness of participants’ own behaviour, to allow them to observe and reflect on the effects of their behaviour on others, and then decide themselves to change their own behaviour. The reflective tasks were the foundation for tailoring the workshop content to the realities and needs of the participants. Thus, most of the material used in the course — such as the examples that were given and discussed, and the demonstrations and role-plays — were based on the submitted self—observation tasks (anonymized). Throughout the workshops it was clear that incorporating these examples, as well as the facilitators sharing their own related experience, was appreciated; participants often laughed when examples were given, commentating that they had done or seen the same, and that it was reassuring to hear of shared issues. This in turn also helped build participants’ ownership of the course and their motivation to learn and make changes to their behaviour:

*“But now, what really was outstanding in this course is you have to do your reflection first. You were given a task to go and reflect yourself. And then now, you come and now you are trained according to your reflections. So, it was more like not a structured training, but they get the training material from the feedback we gave them, so that was what really was outstanding.”* Individual Interview, Nurse, Facility 4
*“The role play one is good. Because it’s the actual thing that happens daily. You know, it was like okay, what you deal with is same with another person. Yeah. The same thing that is happening in hospital XXX is also happening in hospital XXX and is also happening in hospital XXX.”* Individual Interview, Nurse manager, Facility 9

Besides the above examples, most of the nurse managers appreciated an opportunity to come together in the workshop and to share experiences. They applauded the use of role play, demonstrations and group discussions as providing a practical way to better understand the course and the lectures for powerfully bringing together their emerging learning ^23^. Overall, they appreciated the opportunity to connect with eachother:

*“So, when you're discussing with your colleagues, as in in a group, you get so much information and again you connect. The same, same things cut across.”* Individual Interview, Nurse manager, Facility 3

### Recognizing emotional competence as critical to strengthening communication and improving relationships

One of the key aspects of behaviour change was participants realizing and being aware of their own emotions and how these affect people around them as well as recognizing other people’s emotions:

*“You know miscommunication brings a lot of conflicts even between colleagues. But when you are communicating with awareness you as the person you are aware of your own emotions. You are aware of the emotions of your colleagues that you are communicating with. So, you can avoid conflicts. So, what I can say is through this course, I have been able to communicate to let me stay well with my colleagues. We have had less conflict, there is more understanding.”* Individual Interview, Nurse, Facility 1

In particular participants described appreciating, often for the first time, that reactions to situations are often automatic and that greater awareness of those automatic reactions can lead to greater ability to step back in a given situation, to listen actively, to better understand what the issues are, and then to respond with greater awareness. As one participant described:

*A nurse from the nursery mothers ward came to the ward to report a mother who has been absconding from the ward. The NBU nurses had also reported the mother had been missing during night feeding time. Before this course (communication with awareness), this behaviour could have made me very angry but I found myself wanting to explore the reasons for this behaviour. I summoned this mother to my office, stepped back and greeted her and started exploring the reasons why. I explained that absconding from the ward is a serious mistake and that her baby was improving and not going to stay for long in the unit I also explained to her the hospital visiting hours and told her to encourage the husband to visit. She apologised and promised to adhere to what we agreed.”* Nurse, Participant reflection

### Seeing the patient as a person

Many nurses highlighted their new ability to see mothers as people with their own (often significant) social and emotional needs as contributing to better interactions and ultimately relationships with the mothers of the babies in their care. They talked about having a greater understanding of mothers’ frustrations as a result of demonstrating greater respect through listening actively and paying proper attention to what mothers were saying, and through being more patient and less judgemental than they had been in the past. Drawing on their learning on patient-centred care, some described going beyond simply seeking to offer the best treatment to babies, to exploring parents’ inner fears and trying to encourage them:

*“…As a nurse am not just focusing on the sickness, now am doing it wholesomely. Am looking at that mother wholesomely, because rather than the sickness that the mother is having [to deal with], she is a complete mother. She is just like me, she has everything, so now can analyse other faculties her social status, mental status, yes.”* Individual Interview, Nurse, Facility 3
*“…Because now you're more focused on the patient. You're more focused on assisting the patient and making them understand what she is doing and what you want her to do. Unlike just advising her to do this and that. As much as there is advising, you also want to know what she feels or how she takes what you've told her. You asses understanding, you assess- if there's an issue, you can pick it up unlike labelling them. So, you can assist the mothers.*” Individual Interview, Nurse, Facility 1

### Being less judgmental and fearful of colleagues, and building confidence

Greater self-awareness and ability to step back and assess before acting reportedly strengthened nurse managers’ understanding, appreciation and respect of their colleagues. This contributed to their being more likely to consult others, to ask for help and support, and even in some cases to reduce their fear of interacting with more senior colleagues. One interviewee mentioned that she had gained confidence to approach management to mobilize and negotiate for resources, often successfully:

*“…At least they are giving you the support that you require if you ask for anything. If there is an issue you are unable to deal with, when you take it to them, they'll advise you accordingly. Any materials or any, any items that you require in your department, if it is available, they will help you acquire it.”* Individual Interview, Nurse, Facility 3

Relatedly, several managers talked about being much more approachable than they had been in the past, and more willing to help in handling scenarios that would have ignited an emotional exchange within the team in the past. Together with the other impacts described above, these changes appeared to boost job satisfaction and confidence among the nurse managers, and — together with learning about conflict resolution techniques — reportedly reduced the numbers of conflicts and levels of burnout.

*“I observed two nurses who were angrily engaged in a bitter argument about admission of a patient in the ward in front of the baby’s parents. I had to intervene immediately and let the baby be admitted, then had a constructive feedback session with the concerned nurses in regard to their actions and the consequences of their actions and they both understood and accepted their mistake and promised to act differently moving forward.”* Participant Nurse, Reflection task

### Regaining a sense of pride in their profession and being seen by others as a role model

With better relations between colleagues and with family members, a sense of fulfillment and pride in one’s profession appeared to emerge. Nurses reported being re-inspired to think of professional nursing as going beyond clinical tasks to offering wholesome care to patients, and to have new strategies to deal with external stressors that are a hinderance in practicing professional nursing such as burnout and workload:

*“Yes, it is very important, that is why I say it is very difficult to be a professional nurse here. Because I have found this course helping you to deal with the external stressors at your place of work. So, if more of colleagues are involved and trained then you know it becomes easy for everyone to be a key role player when it comes to teamwork and how we treat our patients”.* Individual Interview, Nurse, Facility 4

Several of the nurse managers described that they had changed so much from their old more negative behaviours towards more positive interactions they are now seen as ‘saved’ by colleagues). These nurses felt that their improved awareness and communication skills had strengthened their relationships and reputation among their colleagues, in turn feeding back into their motivation to maintain their new approach:

*The outcome is good, because I came to learn that, when you are a good listener and when you have these good communication skills, you become approachable how you carry yourself, you will find someone coming from a different room, coming to call me. She has skipped two, three or four people on the way, other colleagues. Just to come to me specifically, XXXX, please come and help me to fix this.”* Individual interview, Nurse, Facility 2
*Now most of my colleagues have noticed that I practice a different communication style. I have decided to be a role model so that they can emulate what I do. Some of them have even become conscious of their communication skills while I’m around. To me that means that am making an impact slowly.”* Participant Nurse, Reflection Task

### Limitations in the course approach and impact

Within the positive impacts described above, there were some recommendations for change in the way the course is organised, as well as some limitations in the impact. The issue raised most often was the challenge of managing the self-reflection tasks alongside their normal work and life responsibilities. Almost all participants felt strongly that doing these tasks, including the act of writing down their reflections (as opposed to for example recording them), were important for the reasons described above. However, several felt that there could be less tasks, or that they could be broken down into smaller components with shorter turnaround periods. As two participants explained:

*“For example, if you say for example this week between Monday and Sunday you reflect on this one task and write about it and send. Rather than having several tasks over a period. A period of long time and to me personally, that is what used to give me a little bit of challenges.”* Individual Interview, Nurse, Facility 2
*“Okay, assessing was easier than even thinking of how you will sit, compile, send it, sometimes I used to send late anyway because you are still having lot of work and you still want to practice what you are learning, you want to write the report back, but it required commitment and determination.*” Individual Interview, Nurse, Facility 3.

With regards to sustaining impacts over time, only one and occasionally two people per hospital were included in this course. This appeared to contribute to a feeling of safety and confidence among the group, including to openly share scenarios in the workplace. It also gave the trainer an opportunity to interact in depth with the course participants to explore their areas of strength and weakness and build them up. However to build up teams, and to have a wider and deeper impact, participants felt the training needed to be spread:

*“… you know there are external stressors that I have found this course helping you to deal with, the external stressors at your place of work. So, if more of colleagues are involved and trained then you know it becomes easy for everyone to be a key role player when it comes to team work, how we treat our patients.”* Individual Interview, Nurse manager, Facility 4

Although many participants emphasized the value of the course in dealing with the numerous chronic stressors such as high workloads and limited staffing that they face, some also felt that their new skills were challenging to implement, and even added to the workload. Others reported facing sarcasm from colleagues while practising the skills at work as shared:

*“…It is not difficult per se but I think you see like when you have so many mothers like 80 babies with 80 mothers, trying to empathize with each and every one, you will be worn out, but I don’t, I am not really saying that we are not going to empathize or sympathize but we try.”* Individual Interview, Nurse, facility 7
*“Most of my colleagues have noted that I communicate differently with the mothers. Some of them have openly made fun or been sarcastic. Comments like, “These mothers are stubborn if you entertain them too much” or “How much do you think you will achieve if you want to listen to everyone?” are common. I don’t let these comments get to me and they have not made me slow on practicing my new skills.”* Participant Nurse, Reflection Task

## Discussion

Communication skills and emotional competence training is increasingly recognised as critical for health professionals working in sub-Saharan Africa in improving quality of neonatal care ^7^. The communication and emotional competence course described in this paper is an adaption of a theoretically informed model designed in collaboration with health care professionals over two decades across a range of contexts (see this site for full details on the iCARE-Haaland model). There is some documented experience of incorporating elements of the course into a broader leadership training programme for health managers on the Coast of Kenya ^29^. However, this is the first time the course has been adapted for nurse managers working in NBUs across a range of Kenyan public sector hospitals.

**Figure 2.**
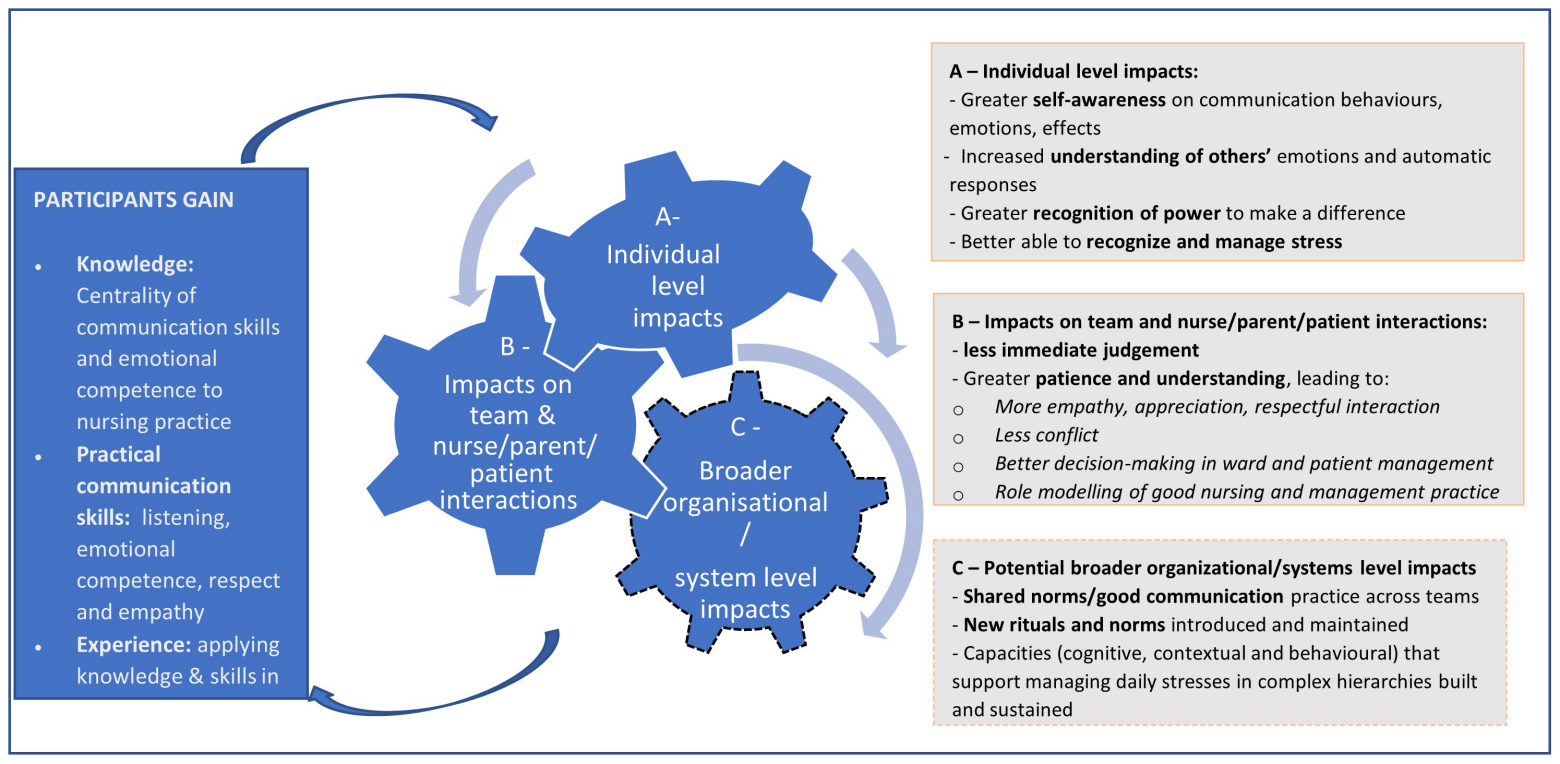
Describes the theory of Change on how the knowledge gained impacts on behavior change at different levels of health system.

Our interviews and observations suggest the participatory training course run over nine months had a wide range of positive impacts for the NBU nurse managers involved, as summarised in the draft theory of change presented in Figure 2. In terms of what participants gained (left box, Figure 2), they gained knowledge on the centrality of communication skills and emotional competence in interactions with patients’ family members and colleagues, and therefore to nursing practice, and on specific issues relevant to their daily work such as conflict resolution, patient/family centred care and human responses to traumas such as death of a child. Participants also reported improved practical skills (including in active listening, management of emotions, and demonstrating respect and empathy), a strong inspiration and motivation to change, and were given opportunities to apply their new knowledge and skills not only in the safe space of the workshop, but also in their normal work places.

Participants described this process as leading to important changes in themselves as individuals including greater awareness of their own and others’ communication behaviours and emotions, and the effects on themselves and others leading to greater confidence and motivation, and a strong sense of professional identity and core values. These reported increases in ‘self-efficacy’ (the belief in your own ability to control your own behaviour, emotions, and motivations), in turn appeared to contribute to better team and patient interactions and relationships ^30^. This appeared to come through greater understanding of their colleagues’ and patient/family members’ situations and concerns, including seeing parents as ‘persons’, reportedly leading to more respectful interactions, fewer conflicts and better decision-making, and in some cases role modelling of good nursing and management practice to teammates. Changes at the intra-personal (Box A) and inter-personal levels (Box B) appeared to feed back into the participants’ gains from the course (left box, Figure 2), further embedding positive change including greater reported job satisfaction and well-being, and reduced levels of stress and feelings of being burnt out. 

These overall positive findings are based on interviews with participants who remained in the training from the outset, and on observations made by the implementing and research team, with limitations as outlined below. However, the literature supports these findings. Studies suggest that increased self-awareness, reflection upon and learning about communication skills can have beneficial effects on health professionals’ relationships with patients and colleagues, and for their own motivation and job satisfaction ^12, 31^. More specifically, nurses’ communication skills training has been shown to increase nurses’ ability to respond to parents’ feelings with empathy ^6^, and nurses with high levels of emotional awareness and control have been shown to communicate more effectively in work encounters ^2, 32^. Three elements appeared to be particularly important to the teaching approach we tracked. Firstly, the use of reflective tasks and discussion of emerging learning contributed to self-awareness, motivation to change, and recognition of shared challenges; an approach which has been supported elsewhere ^33^. Second, it was valuable to have trainers with similar experience to participants as they were able to directly relate to the challenges and contribute their own illustrations and strategies based on having performed similar roles in similar environments. Together with their use of engaging teaching methods (including tailored role-plays and demonstrations) they appeared to create a safe, non-judgmental learning environment. These approaches are recognized in behaviour change theory and adult experience-based learning to motivate individual level change ^34^. Third, and more tentatively, the training was embedded within wider institutional strengthening initiatives linked to the CIN initiative. This may have provided a platform of relationships and activities which positively contributed to nurses’ interest in participating in the communication and emotional competence course, and their ability to act upon and implement new learning, potentially even feeding back into CIN initiatives. 

Of particular interest is that the positive impacts were described by nurse managers working in exceptionally challenging environments, for example where one nurse may have to care for over 15 sick new-borns ^13^. High levels of burnout in such contexts might be better described in some cases as ‘moral injury’, where health workers simultaneously know what care patients need but are unable to provide it due to constraints beyond their control ^35^. Moral injury has potential to feed back into communication challenges with patients and colleagues through contributing to ‘moral neutralisation’, whereby staff become too discouraged to show moral sensitivity, normalize or justify unethical behaviour, or become morally indifferent ^36^. The intensive, stressful and technical environments of public sector new-born units in Kenya, similar to many high dependency settings, raise particular communication-related training needs, as well as challenges for any positive changes to be sustained and spread.

Training senior nurse managers with leadership roles in these environments offers some potential for impacts to be spread and sustained across teams and more widely (Box C in Figure 2). This was hinted at in our data when nurses talked about, for example being (newly) seen as role-models and in better handling work-based conflicts. They may have been introducing new norms and routines around interactions with parents, how conflicts among staff are handled, and how issues are raised and responded to across hierarchical teams and different professions. However, shifting norms in this way *i.e.* ‘the way things are done around here’, is notoriously difficult, and depends to some extent on the formal and informal power and status of the trained nurses among their peers and in relation to other powerful actors in the system, in particular doctors ^21^. Even for powerful individuals within one profession, shifting norms and behaviour in another profession can be particularly difficult. In these ways, sustaining and spreading the potential gains of such training are at risk of being undermined by those same work environment forces that necessitate the training in the first place ^29^. 

The importance of developing a larger and cross-professional critical mass of those with the knowledge, skills and — importantly — the motivation to change was recommended by the course participants. We are currently piloting in two of the hospitals a ‘Trainer’ approach to test the potential of the course to be conducted by the nurse managers to train other nurses within their hospital in an effort to increase sustainability and wider spread of the adapted course. The new trainers will be taught to lead the course with approximately 20 colleagues from each hospital, with the support of the lead trainer of the course described in this paper. This will be one approach aimed at ensuring there is more role modelling and peer support within hospitals, contributing to course gains being sustained and spread. Depending on the outcome of this next phase, further opportunities to sustain and spread the positive impacts of the course include introducing elements of the course into continuous professional education initiatives in hospitals, into CIN activities across a wider network of hospitals, and into undergraduate teaching. As we continue to evolve and track the training through these approaches, it is vital to explore if and how the observed and potential impacts of this course, as outlined in Figure 2, are maintained, sustained and spread across individual, team and system levels. Such evaluations should include perceptions of stakeholders beyond participants and should test some of the assumptions behind Figure 2, including that greater awareness of emotions is positive for those who gain that awareness, and that strengthened awareness and communication behaviour is seen in a positive light by untrained colleagues. 

### Study strengths and limitations

The study was conducted by a research team with deep tacit knowledge of the specific context in which nurses work, and the everyday pressures faced in resource constrained settings. Findings from a range of qualitative methods were triangulated to strengthen trustworthiness of the data and their interpretation, including observation of the course process and activities, individual and group interviews, pre and post course questionnaires, and self-reflections. Nevertheless the main study limitations are interviews only being conducted with course participants, potential hesitation in the interviewees raising concerns about the course process and the data being collected over a relatively short period of time (12 months).

## Conclusions

Our findings suggest that the participatory ‘on the job’ training process described in this paper has potential to have a positive impact on individuals and teams even in significantly resource constrained hospitals. The approach used appeared to strengthen nurses’ awareness of their own, their colleagues’ and their patients’ emotional triggers and responses, and how these influence the nature and the quality of relationships with patients and colleagues. This in turn reportedly influenced their confidence, sense of professional identity, and ability to provide respectful care, including through strengthening their ability to see the patient as a person. There is some potential for positive impacts, at least in theory, to be spread across teams and more widely. However, any such potential is also challenged by the intensive, stressful and technical environments of public sector new-born units in Kenya that contributed to the communication and emotional-management related needs in the first place. Trained nurses highlighted a need for other nurses and cadres in the nurses’ networks to be trained using similar reflection-based and participatory approaches.

## Abbreviations

LMIC-Low- and Middle-income countries

CME-Continuous medical education                      NBU-Newborn unit 

CST-Communication skills training                       CIN-Clinical Information network

iCARE- Intelligent Communication, Awareness and Action, Reflection, Emotions

## Data Availability

The underlying data consists of recordings and transcripts that are confidential and cannot be effectively deidentified because participants were sharing some personal information about themselves and their patients that cannot be shared publicly. However this data (transcripts) will be available on request through filling a data request form found https://dataverse.harvard.edu/file.xhtml?fileId=6359178&version=2.1 or send a request email to dgc@kemri-wellcome.org. Once request form has been shared, the lead author will be able to share the transcripts and any other related data. Harvard Data verse: Strengthening respectful communication with patients and colleagues in neonatal units – Developing and evaluating a communication and emotional competence training for nurse managers in Kenya. https://doi.org/10.7910/DVN/VEZBB1 ^33^ This project contains the following extended data: Interview guide_07112019.pdf (Interview guide used for in-depth interviews) Observational task 1- Communication skills- A self-observation tool on what happens when you communicate well Observation task 2 - Handling emotions-A self-observation tool on what happens when angry or when death happens Observation task 3- Skill into practice -A self-observation of putting the skills learned into practice Pre course questionnaire -Administered before the start of the course to determine needs assessment

## References

[1] Bailey C Murphy R Porock D : Professional tears: developing emotional intelligence around death and dying in emergency work. *J Clin Nurs.* 2011;20(23–24):3364–72. 10.1111/j.1365-2702.2011.03860.x 22017523

[2] Bamberger E Genizi J Kerem N : A pilot study of an emotional intelligence training intervention for a paediatric team. *Arch Dis Child.* 2017;102(2):159–64. 10.1136/archdischild-2016-310710 27737839

[3] Nzinga J McKnight J Jepkosgei J : Exploring the space for task shifting to support nursing on neonatal wards in Kenyan public hospitals. *Hum Resour Health.* 2019;17(1): 18. 10.1186/s12960-019-0352-x 30841900 PMC6404312

[4] Wilkinson S Perry R Blanchard K : Effectiveness of a three-day communication skills course in changing nurses’ communication skills with cancer/palliative care patients: a randomised controlled trial. *Palliat Med.* 2008;22(4):365–75. 10.1177/0269216308090770 18541641

[5] Disch J : Are we *really* ready for patient-centered care? *Nurs Outlook.* 2012;60(5):237–9. 10.1016/j.outlook.2012.07.001 23036752

[6] Bry K Bry M Hentz E : Communication skills training enhances nurses’ ability to respond with empathy to parents’ emotions in a neonatal Intensive Care Unit. *Acta Paediatr.* 2016;105(4):397–406. 10.1111/apa.13295 26648201 PMC5066675

[7] Horwood C Haskins L Luthuli S : Communication between mothers and health workers is important for quality of newborn care: A qualitative study in neonatal units in district hospitals in South Africa. *BMC Pediatr.* 2019;19(1): 496. 10.1186/s12887-019-1874-z 31842824 PMC6913017

[8] Larson E Leslie HH Kruk ME : The determinants and outcomes of good provider communication: a cross-sectional study in seven African countries. *BMJ Open.* 2017;7(6): e014888. 10.1136/bmjopen-2016-014888 28674138 PMC5734554

[9] Little JW : Professional communication and collaboration. *The keys to effective schools: educational reform as continuous improvement*. Second Ed.2007;51–66. 10.4135/9781483329512.n4

[10] Dubale BW Friedman LE Chemali Z : Systematic review of burnout among healthcare providers in sub-Saharan Africa. *BMC Public Health.* 2019;19(1): 1247. 10.1186/s12889-019-7566-7 31510975 PMC6737653

[11] Nzinga J Mbindyo P Mbaabu L : Documenting the experiences of health workers expected to implement guidelines during an intervention study in Kenyan hospitals. *Implement Sci.* 2009;4(1): 44. 10.1186/1748-5908-4-44 19627591 PMC2726115

[12] Kwame A Petrucka PM : Communication in nurse-patient interaction in healthcare settings in sub-Saharan Africa: a scoping review. *Int J Africa Nurs Sci.* 2020;12: 100198. 10.1016/j.ijans.2020.100198

[13] Gathara D Serem G Murphy GAV : Missed nursing care in newborn units: a cross-sectional direct observational study. *BMJ Qual Saf.* 2020;29(1):19–30. 10.1136/bmjqs-2019-009363 31171710 PMC6923939

[14] Orzalesi M Aite L : Communication with parents in neonatal intensive care. *J Matern Fetal Neonatal Med.* 2011;24 Suppl 1(SUPPL. 1):135–7. 10.3109/14767058.2011.607682 21942613

[15] Turner M Chur-Hansen A Winefield H : The neonatal nurses’ view of their role in emotional support of parents and its complexities. *J Clin Nurs.* 2014;23(21–22):3156–65. 10.1111/jocn.12558 24575971

[16] Brown H : Hospital domestics: care work in a Kenyan hospital. *Sp Cult.* 2012;15(1):18–30. 10.1177/1206331211426056

[17] de Sousa Mata ÁN de Azevedo KPM Braga LP : Training in communication skills for self-efficacy of health professionals: a systematic review. *Hum Resour Health.* 2021;19(1): 30. 10.1186/s12960-021-00574-3 33676515 PMC7937280

[18] McGilton K Irwin-Robinson H Boscart V : Communication enhancement: nurse and patient satisfaction outcomes in a complex continuing care facility. *J Adv Nurs.* 2006;54(1):35–44. 10.1111/j.1365-2648.2006.03787.x 16553689

[19] Khodadadi E Ebrahimi H Moghaddasian S : The effect of communication skills training on quality of care, self-efficacy, job satisfaction and communication skills rate of nurses in hospitals of Tabriz, Iran. *J Caring Sci.* 2013;2(1):27–37. 10.5681/jcs.2013.004 25276707 PMC4161104

[20] Mullan BA Kothe EJ : Evaluating a nursing communication skills training course: the relationships between self-rated ability, satisfaction, and actual performance. *Nurse Educ Pract.* 2010;10(6):374–8. 10.1016/j.nepr.2010.05.007 20541974

[21] Foronda C MacWilliams B McArthur E : Interprofessional communication in healthcare: an integrative review. *Nurse Educ Pract.* 2016;19:36–40. 10.1016/j.nepr.2016.04.005 27428690

[22] Nzinga J Jones C Gathara D : Value of stakeholder engagement in improving newborn care in Kenya: a qualitative description of perspectives and lessons learned. *BMJ Open.* 2021;11(6): e045123. 10.1136/bmjopen-2020-045123 34193487 PMC8246352

[23] Branch WT : Teaching professional and humanistic values: suggestion for a practical and theoretical model. *Patient Educ Couns.* 2015;98(2):162–7. 10.1016/j.pec.2014.10.022 25468396

[24] Hulscher ME Laurant MG Grol RP : Process evaluation on quality improvement interventions. *Qual Saf Health Care.* 2003;12(1):40–6. 10.1136/qhc.12.1.40 12571344 PMC1743654

[25] Tuti T Bitok M Paton C : Innovating to enhance clinical data management using non-commercial and open source solutions across a multi-center network supporting inpatient pediatric care and research in Kenya. *J Am Med Inform Assoc.* 2016;23(1):184–92. 10.1093/jamia/ocv028 26063746 PMC4681113

[26] Nzinga J McGivern G English M : Hybrid clinical-managers in Kenyan hospitals: Navigating between professional, official and practical norms. *J Health Organ Manag.* 2019;33(2):173–87. 10.1108/JHOM-08-2017-0203 30950310 PMC6776117

[27] Furber C : Framework analysis: a method for analysing qualitative data. *Afr J Midwifery Womens Health.* 2010;4(2):97–100. 10.12968/ajmw.2010.4.2.47612

[28] Gale NK Heath G Cameron E : Using the framework method for the analysis of qualitative data in multi-disciplinary health research. *BMC Med Res Methodol.* 2013;13: 117. 10.1186/1471-2288-13-117 24047204 PMC3848812

[29] Nzinga J Boga M Kagwanja N : An innovative leadership development initiative to support building everyday resilience in health systems. *Health Policy Plan.* 2021;36(7):1023–1035. 10.1093/heapol/czab056 34002796 PMC8359752

[30] Nørgaard B Ammentorp J Ohm Kyvik K : Communication skills training increases self-efficacy of health care professionals. *J Contin Educ Health Prof.* 2012;32(2):90–7. 10.1002/chp.21131 22733636

[31] Raeissi P Zandian H Mirzarahimy T : Relationship between communication skills and emotional intelligence among nurses. *Nurs Manag (Harrow).* 2019;26(2):31–35. 10.7748/nm.2019.e1820 31468761

[32] Amini M Amini M Nabiee P : The relationship between emotional intelligence and communication skills in healthcare staff. *Shiraz E Med J.* 2019;20(4): e80275. 10.5812/semj.80275

[33] Mann K Gordon J MacLeod A : Reflection and reflective practice in health professions education: a systematic review. *Adv Health Sci Educ Theory Pract.* 2009;14(4):595–621. 10.1007/s10459-007-9090-2 18034364

[34] Joyce JP Pfau RG Berman IJ : Facial burning and scarring in a child. *Arch Dermatol.* 1985;121(7):928, 931. 10.1001/archderm.121.7.928 4015142

[35] Dean W Talbot S Dean A : Reframing clinician distress: moral injury not burnout. *Fed Pract.* 2019;36(9):400–2. 31571807 PMC6752815

[36] Hakimi S Hejazi E Lavasani MG : The relationships between personality traits and students’ academic achievement. *Procedia - Soc Behav Sci.* 2011;29:836–45. 10.1016/j.sbspro.2011.11.312

