# Supplementary material for: Strengthening respectful communication with patients and colleagues in neonatal units — developing and evaluating a communication and emotional competence training for nurse managers in Kenya
Source: Wellcome Open Res. 2025 Apr 25;7:223. Originally published 2022 Sep 5. [Version 2] doi: 10.12688/wellcomeopenres.18006.2 (PMC11066535; doi:10.12688/wellcomeopenres.18006.2)
Supplement: Peer review history

# Reviewer report: Reviewer response for version 1

**DOI:** 10.21956/wellcomeopenres.19965.r74000

**Published:** 2024-05-02

**Authors:** Laura Dean

**Affiliations:** 1 Liverpool School of Tropical Medicine, Liverpool, England, UK

This is a well written and compelling article that addresses a key area in health workforce skills development. The participatory adaptation and delivery of a training intervention to enhance communication and emotional competence among nurses in neonatal care is presented. The training was positively evaluated and the potential of such and intervention clearly articulated. 

 The clarifications or questions that arose for me were minimal, as follows: 

 Although the sample is relatively homogeneous, I was interested to understand if there were other characteristics that altered the impact of the intervention. For example, length of service, age, previous personal maternal health experiences, job role prior to the neonatal unit. The complexities of work related trauma and the impact of such interventions in relation to some of these characteristics may be interesting to explore in future work. Where possible, nuance in experience would also be beneficial to consider within the presentation of results here. 

 I noticed approximately 4 individuals did not complete the end line but did complete baseline data collection activities- what was the reason for this? did they not complete the course? if so why not? This is important to also understand in light of the positive findings to ensure retention in future endeavours. 

 Overall, i think this is a useful article, not just in terms of supporting improvements in care within neonatal units, but with significant potential for other situations in resource constrained environments.

Is the work clearly and accurately presented and does it cite the current literature?

Yes

If applicable, is the statistical analysis and its interpretation appropriate?

Not applicable

Are all the source data underlying the results available to ensure full reproducibility?

Yes

Is the study design appropriate and is the work technically sound?

Yes

Are the conclusions drawn adequately supported by the results?

Yes

Are sufficient details of methods and analysis provided to allow replication by others?

Yes

Reviewer Expertise:

Participatory Health Research

I confirm that I have read this submission and believe that I have an appropriate level of expertise to confirm that it is of an acceptable scientific standard.

# Response

**Published:** 2025-03-27

**Authors:** Sassy Molyneux

**Affiliations:** University of Oxford, UK

We are very grateful for the positive and careful reviews of our paper, and for the very useful suggestions to strengthen it.  We attach a revised copy of the paper, with the changes highlighted in yellow.  A point by point response to each comment from the reviewers is provided below. Reviewers' comments: This is a well written and compelling article that addresses a key area in health workforce skills development. The participatory adaptation and delivery of a training intervention to enhance communication and emotional competence among nurses in neonatal care is presented. The training was positively evaluated and the potential of such and intervention clearly articulated. 

 The clarifications or questions that arose for me were minimal, as follows: 

**Response – thank you**

 Although the sample is relatively homogeneous, I was interested to understand if there were other characteristics that altered the impact of the intervention. For example, length of service, age, previous personal maternal health experiences, job role prior to the neonatal unit. The complexities of work related trauma and the impact of such interventions in relation to some of these characteristics may be interesting to explore in future work. Where possible, nuance in experience would also be beneficial to consider within the presentation of results here. 

**Response – we agree this is very important and would have been valuable to explore in more depth as part of our analyses.  We will ensure we do so in future on-going related work.**

 I noticed approximately 4 individuals did not complete the end line but did complete baseline data collection activities- what was the reason for this? did they not complete the course? if so why not? This is important to also understand in light of the positive findings to ensure retention in future endeavours. 

**Response – we have now provided information on this (see under reviewer 1)**

 Overall, i think this is a useful article, not just in terms of supporting improvements in care within neonatal units, but with significant potential for other situations in resource constrained environments.

**Response – thank you**

# Reviewer report: Reviewer response for version 1

**DOI:** 10.21956/wellcomeopenres.19965.r58404

**Published:** 2023-07-28

**Authors:** Anna P Ralph

**Affiliations:** 1 Global and Tropical Health, Menzies School of Health Research, Charles Darwin University, Casuarina, Northern Territory, Australia

**Content**

 This is an important study addressing a fundamental clinical skill (communication) which is often taught poorly or not at all. The authors developed and implemented training on communication and emotional competence, delivered over 9 months for nurses working in neonatal care in 14 Kenyan hospitals. The training was very favourably received, and successful in eliciting improved self-reported communication styles among the trainees. Methods are sound, data are presented clearly and the conclusions are appropriate. The following questions arose in my reading of the paper.  

 From what I can gather, trainees met face to face with the trainer 3 times, the first being a situation analysis and the next two comprising actual training days, with a 5 month gap between the second and final workshop. How was momentum maintained in the interim? Did trainees receive feedback on their reflections that they submitted and if so, how was that provided? (phone, email). The intensity of the intervention seems ‘light’ and yet the impact seems large in that benefits of the training are well described – were there components to the training that are not clearly coming through in the methodological description?

 What was the retention in the course over the 9 months? How many trainees dropped out?

 What language were interviews conducted in? If English, was that appropriate?

 Interviews were done by a researcher independent from the one who ran the training. However to what extent do you think interview participants could speak honestly about their experience of the course, given that they knew the interviewee was a researcher on the project?

 Why are there such power differentials between nurses and patients in this context – e.g. the comment “These mothers are stubborn if you entertain them too much” demonstrates 'othering' are the mothers largely a different ethnic group, or are from a different socioeconomic stratum, from the staff. What do those differences comprise, and were they specifically addressed in the training?

 Why was the choice made to select 1-2 staff per hospital, rather than a larger cohort from a smaller number of hospitals? I imagine that impact could be diluted if there are few attendees per site, whereas a larger cohort from fewer sites might help achieve the goal of cultural change within hospitals as more staff learn and can then implement their new skills together and positively reinforce behaviour?

 In the Theory of Change, C indicates that there was diffusion of learning that occurred beyond those who attended the training – but is much evidence presented for that in this paper? (only course participants were interviewed). One participant said they hoped others might emulate their new communication style, but were there efforts made to capture whether there was in fact broader benefit beyond those staff who attended the training?

**Typographical errors and spelling**

 Table 1 contains this question “Week 1: Listening: How well do you listen to your patient’s colleagues, others and effects?”

 Should this read: Week 1: Listening: How well do you listen to your patients, colleagues and others?

 Phase 4 c reads “c) Why and how to recognize and manage, and prevent stress and burnout” should probably read c) Why and how to recognize, manage and prevent stress and burnout

 Methods – in the sentence “…until the researchers found that no new themes were not being introduced”, remove ‘not’.

 In the sentence “Beyond the specific elements of the course feeding into change as outline next”, change outline to outlined

 Behaviour is spelled variously with or without the ‘u’ – please edit for consistency.

 The quotes are transcripts from interviews so I believe punctuation and spelling errors can be corrected? E.g. “follow-ng” in the first quote and “hav-ng” in the second quote should be corrected

 “Me my most…” (comma after me?)

 “Some of them have even become conscious of their communication skills while am around.” Replace am with I’m? (transcription error?)

 “...observations made the implementing and research team” – add ‘by’ after ‘made’.

Is the work clearly and accurately presented and does it cite the current literature?

Yes

If applicable, is the statistical analysis and its interpretation appropriate?

Not applicable

Are all the source data underlying the results available to ensure full reproducibility?

Yes

Is the study design appropriate and is the work technically sound?

Yes

Are the conclusions drawn adequately supported by the results?

Yes

Are sufficient details of methods and analysis provided to allow replication by others?

Yes

Reviewer Expertise:

Tropical infectious diseases, First Nations health and inter-cultural communication

I confirm that I have read this submission and believe that I have an appropriate level of expertise to confirm that it is of an acceptable scientific standard.

# Response

**Published:** 2025-03-27

**Authors:** Sassy Molyneux

**Affiliations:** University of Oxford, UK

We are very grateful for the positive and careful reviews of our paper, and for the very useful suggestions to strengthen it.  We attach a revised copy of the paper, with the changes highlighted in yellow.  A point by point response to each comment from the reviewers is provided below:

 This is an important study addressing a fundamental clinical skill (communication) which is often taught poorly or not at all. The authors developed and implemented training on communication and emotional competence, delivered over 9 months for nurses working in neonatal care in 14 Kenyan hospitals. The training was very favourably received, and successful in eliciting improved self-reported communication styles among the trainees. Methods are sound, data are presented clearly and the conclusions are appropriate. The following questions arose in my reading of the paper.  

**Response – thank you**

 From what I can gather, trainees met face to face with the trainer 3 times, the first being a situation analysis and the next two comprising actual training days, with a 5 month gap between the second and final workshop. How was momentum maintained in the interim? Did trainees receive feedback on their reflections that they submitted and if so, how was that provided? (phone, email). The intensity of the intervention seems ‘light’ and yet the impact seems large in that benefits of the training are well described – were there components to the training that are not clearly coming through in the methodological description?

**Response – this is a really helpful clarification.  There was regular informal interactions on the phone primarily via whatsapp, when the trainers wanted input and support from the lead trainer.  Reflections were submitted via whatsapp/ email to the training lead, but they were not individually discussed outside of the training days.   We have now added this information into the methodological description.**

 What was the retention in the course over the 9 months? How many trainees dropped out? **Response – of the 18 in-charges who began the course, two shifted departments and so 16 completed the entire process..** **We have now added this information into the methodological description.**

 What language were interviews conducted in? If English, was that appropriate?

**Response – the interviews were in English or Kiswahili, as preferred by the participants.  We have now added this information into the methodological description.**

 Interviews were done by a researcher independent from the one who ran the training. However to what extent do you think interview participants could speak honestly about their experience of the course, given that they knew the interviewee was a researcher on the project?

**Response – there were some critiques of the course as outlined in the paper, but there is certainly some potential for interviewees to be hesitant in giving their honest views.  We have now specifically noted this in the limitations section near the end of the discussion.**

 Why are there such power differentials between nurses and patients in this context – e.g. the comment “These mothers are stubborn if you entertain them too much” demonstrates 'othering' are the mothers largely a different ethnic group, or are from a different socioeconomic stratum, from the staff. What do those differences comprise, and were they specifically addressed in the training?

**Response – There is some othering of mothers by staff, partly as a coping strategy with their high workloads, and potentially linked to many mothers being from different ethnic groups and of lower SES than the nurses.  This was indeed a central element of the training as part of maintaining dignity and respect and changing attitudes.  We have not added this detail only because there is so much more detail we could have also added, but it’s available in the longer training material to interested readers.**

 Why was the choice made to select 1-2 staff per hospital, rather than a larger cohort from a smaller number of hospitals? I imagine that impact could be diluted if there are few attendees per site, whereas a larger cohort from fewer sites might help achieve the goal of cultural change within hospitals as more staff learn and can then implement their new skills together and positively reinforce behaviour?

**Response – the staff who were included were the in-charges of the NBUs, with all who were involved in the CIN network being given the opportunity to be included.  The in-charges were targeted given the importance of mid-level managers/leaders to the functioning of their teams.  This had always been planned as the first step in a cascading of the training to more people within each facility through a TOT process.  Experiences with the latter is now being written up and submitted as a separate paper.  We have now noted this in the methodological description and the discussion section.**

 In the Theory of Change, C indicates that there was diffusion of learning that occurred beyond those who attended the training – but is much evidence presented for that in this paper? (only course participants were interviewed). One participant said they hoped others might emulate their new communication style, but were there efforts made to capture whether there was in fact broader benefit beyond those staff who attended the training?

**Response – the C in the ToC is tentative, based on the hope that this diffusion of learning might happen, especially with the cascading of care described above.  We have emphasised how tentative this is in the discussion and Theory of Change.  In our next paper soon to be submitted for publication, we have included perspectives from non-trained colleagues and managers.**

**Typographical errors and spelling – thank you; all nine have been corrected.**
